# Trapezoidal resection of an elongated anterior mitral leaflet and Alfieri stitch in hypertrophic cardiomyopathy

**DOI:** 10.1186/s13019-020-01361-2

**Published:** 2020-10-12

**Authors:** Hiroyuki Nakajima, Chiho Tokunaga, Jun Hayashi, Akitoshi Takazawa, Akihiro Yoshitake, Atsushi Iguchi

**Affiliations:** Department of Cardiovascular Surgery, Saitama Medical University, International Medical Center, 1397-1 Yamane Hidaka, Saitama, 350-1298 Japan

**Keywords:** Hypertrophic obstructive cardiomyopathy, Mitral valve repair, Systolic anterior motion, Septal hypertrophy, Myectomy

## Abstract

**Background:**

In individuals with hypertrophic obstructive cardiomyopathy, elongated anterior mitral leaflets are commonly associated with systolic anterior motion. In patients with mild septal hypertrophy, a myectomy is considered insufficient to relieve systolic anterior motion and left ventricular outflow tract obstruction.

**Case presentation:**

In the patient, who had relatively mild septal hypertrophy, the section of the anterior leaflet protruding into the left ventricular outflow tract was resected, concomitant with septal myectomy and the relocation of the papillary muscles. An edge-to-edge stitch was placed at the uppermost segment of the coaptation zone. Using these manoeuvres, systolic anterior motion, left ventricular outflow tract obstruction and mitral regurgitation were successfully resolved postoperatively.

**Conclusions:**

We describe a surgical technique with an edge-to-edge suture for the resection of an elongated anterior mitral leaflet. In combination with septal myectomy and relocation of the papillary muscles, this technique is a simple and viable option, especially when septal hypertrophy is not severe.

## Background

Structural mitral valve abnormalities are frequently observed in individuals with hypertrophic cardiomyopathy. Redundant or elongated anterior mitral leaflets are commonly associated with systolic anterior motion (SAM). Here, we describe a simple surgical technique for the removal of an elongated anterior mitral leaflet.

## Case presentation

**Fig. 1:**
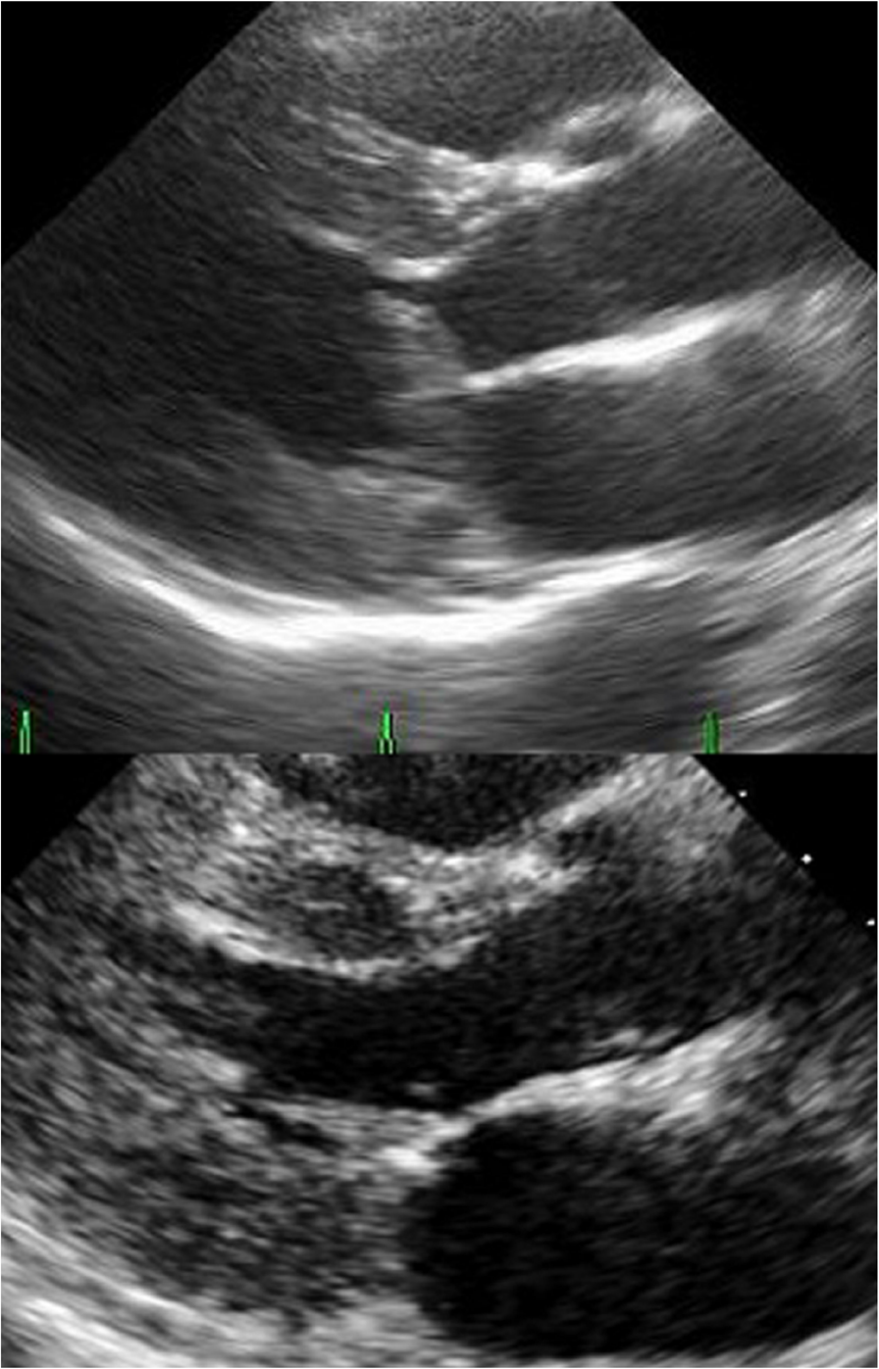
(Top) Preoperative transthoracic echocardiography revealed a left ventricular outflow obstruction caused by systolic anterior motion of the anterior mitral leaflet. The septal thickness was 17 mm at the contact point. (Lower) Postoperatively, echocardiography showed the absence of systolic anterior motion and mitral regurgitation. Accelerated flow in the left ventricular outflow tract was not detected

**Fig. 2:**
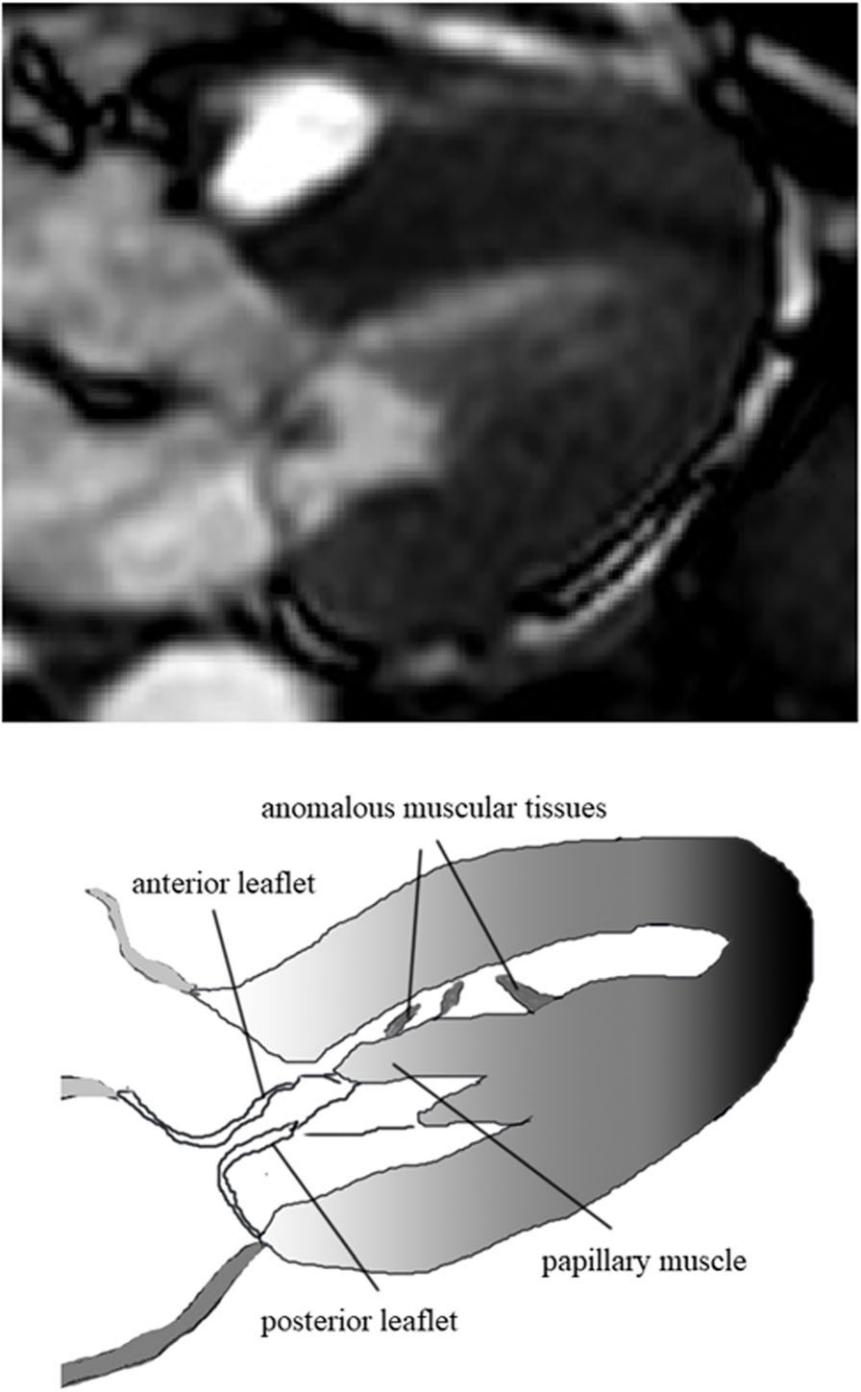
Cardiac magnetic resonance imaging in late systole. In this patient, two mechanisms of SAM were postulated. One was that the tips of papillary muscles anomalously deviated anteriorly. The papillary muscles were connected with the septum with abnormal fibrous tissue and touching the septum in systole. The other mechanism involves the elongated anterior leaflet. It was turned over by the blood flow through LVOT, as described by Schwammenthal et al. [1]

A 76-year-old woman with exertional dyspnoea was referred to our hospital. Echocardiography revealed the presence of SAM and severe mitral regurgitation (MR). The left ventricular diastolic dimension and ejection fraction were 39 mm and 81%, respectively. The elongated A2 segment contacted the septum at systole, causing left ventricular outflow tract obstruction (LVOTO) (Fig. 1, top). Although oral medication was administered, the left ventricular outflow tract (LVOT) gradient was determined to be 127 mmHg. Septal hypertrophy was relatively mild. The maximum thickness of the septum beneath the aortic annulus was 17 mm. The pressure gradient of tricuspid regurgitation was 21 mmHg. The functional status was classified as New York Heart Association class II. Significant stenosis was not observed in the coronary or carotid arteries. The patient did not have a history of syncope or ventricular arrhythmia. Cardiac magnetic resonance imaging revealed that the papillary muscle tips had deviated anomalously to the anterior or septal walls due to the attachment of fibrous or muscular tissues between the septum and the papillary muscles (Fig. 2). Although septal ablation was considered, a reduction in the septal thickness alone was deemed to be insufficient to relieve SAM. Taking the case of severe LVOTO and the risk of sudden death into account, we decided to first perform surgical repair.

**Fig. 3:**
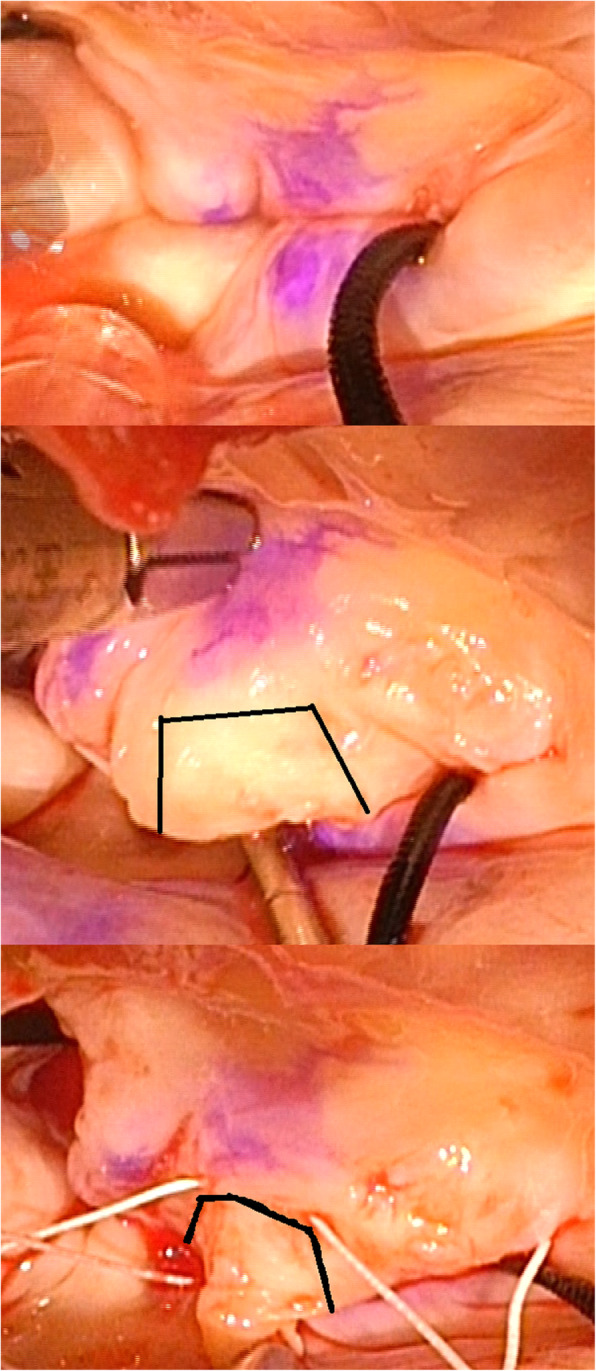
After subvalvular manoeuvres, the coaptation of A2 and P2 was evaluated using a blue marker (top). A 5–0 expanded polytetrafluoroethylene horizontal mattress suture was placed, with the elongated portion of the anterior leaflet below the stitch (middle). The segment (solid line) with the attached chorda was resected (middle and bottom). Finally, the ePTFE stitch was tied, and the mitral valve remained competent

Through a right-sided left atriotomy, the mitral valve was inspected. Thickened secondary chordae and muscular bundles connecting the papillary muscles and the anterior or septal wall were divided. Both papillary muscles were repositioned posteriorly by fixing the secondary papillary muscle with 4–0 expanded polytetrafluoroethylene (ePTFE) mattress sutures. The competency of the mitral valve was then confirmed (Fig. 3, top). The A2 segment was thickened and anomalously elongated and was considered the cause of the SAM (Fig. 3, middle). A 5–0 ePTFE horizontal mattress suture was placed at the uppermost segment of the coaptation zone. Before the suture was tied, a trapezoidal portion of the elongated leaflet below the ePTFE stitch and the attached chordae were resected (12 × 12 mm) (Fig. 3, bottom). Subsequently, a 6 × 6 mm trapezoidal section of the posterior leaflet below the suture was resected. Finally, the edge-to-edge ePTFE suture was tied.

Transverse aortotomy was performed just above the sinotubular junction. Myectomy was performed through the aortic valve. Due to mild hypertrophy, myectomy was performed carefully in a section measuring approximately 10 mm in width and < 5 mm in thickness.

After the patient was weaned from cardiopulmonary bypass, transoesophageal echocardiography revealed the absence of accelerated flow and MR; the next morning, she was extubated. The postoperative course was uneventful. Transthoracic echocardiography revealed that LVOTO, MR and SAM were not detected (Fig. 1, bottom). The patient was discharged from the hospital on postoperative day 12. The symptoms disappeared, and there were no ventricular arrhythmia or cardiac events postoperatively. The automated implantable cardioverter defibrillator has not yet been implanted. At 2.5 years after the operation, with echocardiography, SAM was not seen. Trivial mitral regurgitation was detected, and the peak pressure gradient through LVOT was calculated to be 14 mmHg.

The resection of the anterior mitral leaflet and the placement of an edge-to-edge stitch, concomitant with septal myectomy and the relocation of papillary muscles, were performed in four patients (Table 1). In one patient (case 5), the manoeuvres were only performed on the anterior leaflet to treat SAM associated with mitral valve repair. In all cases, SAM, MR and LVOTO were successfully resolved. In one patient (case 4) with a small left ventricle and a diastolic dimension of 30 mm, a new accelerated flow at the mid-ventricle was detected, probably due to the Venturi effect.

**Table 1:** Procedures performed and echocardiographic data

	Age	Sex	Diagnosis and echocardiographic findings	Preoperative measures			Procedures performed			Latest echocardiography findings		
				Maximum thickness in septum (mm)	MR	PG (mmHg)	AML resection and Alfieri	PM relocation	Septal myectomy	MR	PG (mmHg)	Postoperative period
1	76	F	HOCM MR SAM	17	severe	127	Yes	Yes	Yes	trace	14	2.5 years
2	82	F	HOCM MR SAM	22	severe	102	Yes	Yes	Yes	trace	9	1.5 years
3	82	F	HOCM MR SAM	20	severe	93	Yes	Yes	Yes	mild	13	4 months
4	85	F	HOCM MR SAM	22	severe	175	Yes	Yes	Yes	trace	53 (at mid-ventricle)	1 month
5	76	F	SAM after mitral valve repair	20	severe	–	Yes	No	No	mild	29	3 months

*AML* Anterior mitral leaflet, *HOCM* Hypertrophic obstructive cardiomyopathy, *MR* Mitral regurgitation, *PG* Pressure gradient, *PM* Papillary muscle, *SAM* Systolic anterior motion

## Discussion and conclusion

Operative techniques for hypertrophic obstructive cardiomyopathy usually consist of a manoeuvre or a combination of manoeuvres involving the septum, the mitral leaflet and the subvalvular apparatus. When septal hypertrophy is relatively mild, manoeuvres involving the leaflet and the subvalvular apparatus may be crucial. Regarding the manoeuvre involving the leaflet, when the anterior leaflet is elongated by more than 30 mm, plication can shorten the leaflet length by 2–5 mm [2, 3]. The edge-to-edge technique is an option for manoeuvring the mitral leaflet [4, 5]. Currently, this procedure can also be performed on a beating heart [6]. Regarding the manoeuvre on the subvalvular apparatus, chordal cutting was reported to be effective for LVOTO relief through geometric modifications [7]. Moreover, Balaram and colleagues performed a combination of septal myectomy, the release of papillary muscles, and plication of the anterior leaflet [3]. Our technique was a modification of this combination.

Since our patient had relatively mild septal hypertrophy, the abnormal subvalvular apparatus and elongated anterior leaflet played definitive roles in the manifestation of SAM [1]. The papillary muscles were freed from the septal or anterior walls and relocated posteriorly. With our technique involving the mitral leaflet, trapezoidal resection of the elongated anterior leaflet is crucial. The segment of the anterior leaflet that protruded into the LVOT was resected with the chordae. The elongated posterior leaflet was resected in the same manner. After resection, an edge-to-edge suture was essential to maintain valve competence because the coaptation zone became minimal and did not have any chordae attached. The follow-up echocardiography findings revealed that SAM, LVOTO and MR had been successfully resolved for 2.5 years.

In conclusion, resection of the anterior mitral leaflet that causes SAM concomitant with the placement of an edge-to-edge suture is a viable option for treating the leaflet, especially when ventricular hypertrophy is not severe.

## Data Availability

The datasets used or analysed during the current study are available from the corresponding author, on reasonable request.

## Abbreviations

ePTFE: Expanded polytetrafluoroethylene
LVOT: Left ventricular outflow tract
LVOTO: Left ventricular outflow tract obstruction
MR: Mitral regurgitation
SAM: Systolic anterior motion
